# *Aspergillus niger* Infection of an Orbital Exenteration Socket Can Be Treated with Oral Itraconazole

**DOI:** 10.1155/2012/763651

**Published:** 2012-12-24

**Authors:** Wing Lung Alvin So, Thomas G. Hardy

**Affiliations:** Orbital, Plastics and Lacrimal Clinic, Royal Victorian Eye and Ear Hospital, 32 Gisborne Street, East Melbourne, VIC 3002, Australia

## Abstract

*Aspergillus niger* infection is a rare complication following orbital exenteration, especially in immunocompetent patients. If untreated, the infection may lead to significant morbidities. We report a patient with this rare infection, who has been treated successfully with oral itraconazole.

## 1. Introduction

*Aspergillus niger* is usually found in soil and decaying plants, and its infection is uncommon in humans. We report a rare complication of *Aspergillus niger* infection in the orbital exenteration socket in an immunocompetent patient. 

## 2. Case Report

An 89-year-old man presented in November 2006 with a red, watery, sticky left eye, and an enlarging lump (approximately 8 mm at presentation) in the left medial canthus which had been slowly progressive over the last few years. His past history was unremarkable, and in particular, he had no past history of cancer or immunosuppression. His visual acuity was 6/9 (right) and 6/12 (left). There were no palpable lymph nodes. He subsequently underwent an incisional biopsy of the left medial canthus which revealed extensive infiltration by poorly differentiated basi-squamous carincoma. There was no evidence of orbital invasion on computerised tomography (CT). Mohs micrographic surgery was undertaken by a dermatologist, though the deep margin in the medial canthus could not be cleared. He underwent further excision with intraoperative frozen section control to achieve clear margins, and medial canthoplasty with periosteal flaps, buccal mucous membrane graft, and a glabellar skin flap. 

Eleven months later there was a recurrence in the left medial canthus. Orbital CT showed no mass lesion, but paranasal sinusitis was noted in the right ethmoid, right frontal, and bilateral sphenoid sinuses at the time. An orbital exenteration was performed with split skin graft repair, and the tumour was clear of all margins. There was a small surgical defect in the ethmoid bone; the frontal nerve was sacrificed. Six weeks later there was incomplete healing of the socket with moderate discharge but no cellulitis and no sino-orbital fistula, and a swab of the socket showed heavy growth of *Staphylococcus aureus* with polymorphonuclear neutrophils. The patient was treated with cephalexin, to which the organism showed sensitivity. At 12 weeks after operation, the patient developed bleeding and discharge from the eye socket, as well as mild cellulitis in the cheek. However, there were no systemic signs of infection. Swab revealed no bacterial growth, but *Aspergillus niger* was isolated. The socket was dressed with silver-based gauze dressing 2-3 times per week, and after consultation with the microbiologist and infectious disease physicians, itraconazole was commenced. Granulation tissue was present one month later, and no further fungus was detected on repeat swabs (April and August 2008, February, July, and September 2009 and March 2010), although Methicillin sensitive *Staphylococcus aureus* was isolated on a number of occasions. The patient remained well although the socket was slow to fully epithelialise, and he underwent repeat split skin grafting to the exenteration socket (October, 2008), and a superficial temporal artery island flap (June, 2010) to promote epithelialisation. 

## 3. Discussion

Although infection is an uncommon complication following orbital exenteration, it may lead to serious complications such as sepsis and encephalitis if untreated [1]. In addition, orbital infection from *Aspergillus niger* in an immunocompetent person is even more uncommon and has only been reported three times in the literature [2–4]. As with other fungal infections, the general perception of *Aspergillus niger* infection is that it only tends to occur in immunocompromised patients [5, 6], for example, haematological malignancy [7, 8], steroid use [9], and diabetes [10]. None of these factors were present in our patient.

*Aspergillus niger* prefers to grow in a moist and warm environment and is commonly found in soil and plants. Hospital kitchens have also been reported as a source of this fungus [11]. It rarely causes human infection, though skin [12] and soft tissue infection [13], tracheal-oseophageal fistula [14], onychomycosis [15], and mycotic aneurysm [16] have been reported. A foreign body, such as prosthesis, may act as a culture medium for this organism. However, our patient was not yet fitted with any osseo-integrated implants. Postoperative treatment with cephalexin might have allowed the opportunistic infection of *Aspergillus niger*. Although preoperative CT suggested paranasal sinusitis, there was no evidence of sino-orbital fistula to explain for the fungal infection. The source of *Aspergillus niger* infection in our patient remains unknown.

Treatment options of orbital *Aspergillus niger* infection include surgical debridement and intravenous amphotericin B. In addition, local irrigation of amphotericin B combined with oral itraconazole has been described [2], which may be the best treatment as it avoids the systemic adverse effects of amphotericin B, particularly in the elderly patients or those with renal impairment. Our patient responded well to oral itraconazole, thus saving the resources to set up a twice daily antimicrobial irrigation regimen. Unfortunately, itraconazole is not subsidised by the Australian government for this indication.

In conclusion, we have reported a rare case of orbital infection by *Aspergillus niger* following exenteration in an immunocompetent patient. The patient responded well to oral itraconazole alone, thus avoiding adverse effects from treatment with other antibiotics and surgery.
